# Redox proteomics of PANC-1 cells reveals the significance of HIF-1 signaling protein oxidation in pancreatic ductal adenocarcinoma pathogenesis

**DOI:** 10.1186/s12967-024-05068-z

**Published:** 2024-03-16

**Authors:** Chaochao Tan, Lichun Chen, Xiaoyu Guan, Wenyi Huang, Yinhong Feng, Ziyi Li, Ling Wu, Xiangping Huang, Qianhui Ouyang, Sixiang Liu, Ying Huang, Jiliang Hu

**Affiliations:** 1 Department of Clinical Laboratory, Hunan Provincial People’s Hospital (The First Affiliated Hospital of Hunan Normal University), Changsha, 410005 China; 2 Tumor Immunity Research Center of Hunan Provincial Geriatric Institute, Hunan Provincial People’s Hospital (The First Affiliated Hospital of Hunan Normal University), Changsha, 410005 China; 3 School of Pharmaceutical Sciences, Guangzhou University of Chinese Medicine, Guangzhou, 510006 China; 4 Department of Emergency, The First Affiliated Hospital of Hunan Normal University (Hunan Provincial People’s Hospital), Changsha, 410006 Hunan China; 5 Institute of Life Sciences, Chongqing Medical University, Chongqing, 400032 China

**Keywords:** Protein cysteine oxidation, iodoTMT, Redox proteomics, Pancreatic ductal adenocarcinoma, PANC-1

## Abstract

**Background:**

Protein cysteine oxidation is substantially involved in various biological and pathogenic processes, but its implications in pancreatic cancer development remains poorly understood.

**Methods and results:**

In this study, we performed a global characterization of protein oxidation targets in PDAC cells through iodoTMT-based quantitative proteomics, which identified over 4300 oxidized cysteine sites in more than 2100 proteins in HPDE6c7 and PANC-1 cells. Among them, 1715 cysteine residues were shown to be differentially oxidized between HPDE6c7 and PANC-1 cells. Also, charged amino acids including aspartate, glutamate and lysine were significantly overrepresented in flanking sequences of oxidized cysteines. Differentially oxidized proteins in PANC-1 cells were enriched in multiple cancer-related biological processes and signaling pathways. Specifically, the HIF-1 signaling proteins exhibited significant oxidation alterations in PANC-1 cells, and the reduced PHD2 oxidation in human PDAC tissues was correlated with lower survival time in pancreatic cancer patients.

**Conclusion:**

These investigations provided new insights into protein oxidation-regulated signaling and biological processes during PDAC pathogenesis, which might be further explored for pancreatic cancer diagnosis and treatment.

**Supplementary Information:**

The online version contains supplementary material available at 10.1186/s12967-024-05068-z.

## Introduction

Pancreatic cancer is featured by rapid pathogenic progression and poor prognosis, which is still one leading cause of cancer-related death worldwide. The doubling of global diagnosis rate for pancreatic cancer during the past two decades further highlighted its significant impact on global health [1, 2]. Pancreatic ductal adenocarcinoma (PDAC) is the predominant subtype of pancreatic cancer, characterized by a 5-year survival rate of under 10%. This poor prognosis is partially attributed to the diagnosis of most cases at advanced stages, resulting from a scarcity of early symptoms and the lack of effective early detection strategies [3, 4]. Multiple treatment strategies, including the combination cytotoxic chemotherapy and recent targeted therapies, have been utilized for pancreatic cancer management in recent years. However, the overall survival for patients with PDAC, particularly those with metastatic diseases, still remains shorter than 12 months [3, 5]. Currently, molecular alterations that drive cancer pathogenesis, including genomic mutations and protein translational modification (PTM) changes, have been viewed as valuable resource for identifying new targetable biomarkers [3, 6–8]. However, there is still an urgent need for a comprehensive understanding of pancreatic cancer biology in order to develop novel targets for PDAC early detection and precision treatment.

Protein cysteine oxidation refers to the set of oxidative PTMs occurring on the thiol group of cysteine side-chains, which could act as a rapid way of regulating protein functions in response to cellular signaling status [9]. In general, protein oxidative modification can be classified into two types: the reversible and irreversible oxidation. Protein reversible oxidation includes protein *S*-nitrosylation [SNO], *S*-glutathionylation [S-SG], *S*-sulfenylation [SOH] and disulfide bonds [S–S], while and irreversible oxidation encompasses Cys sulfinic [Cys-SO2H] and Cys sulfonic [Cys-SO3H] [10, 11]. These redox modifications, which could be triggered by reactive oxygen species (ROS) and other reactive species, have the potential to regulate redox signaling and diverse biological processes [9, 10, 12, 13]. For instance, the *S*-nitrosylation of endonuclease inositol-requiring protein 1α (IRE1α), one key regulator of unfolded protein response (UPR), is critically involved in endoplasmic reticulum dysfunction during heart failure with preserved ejection fraction (HFpEF) [14, 15]. Recent research also demonstrated that the *S*-nitrosylation of GAPDH substantially contributes to the increased tau acetylation during Alzheimer's disease (AD) pathogenesis linked with traumatic brain injury (TBI) [16]. The *S*-glutathionylation of giant elastic protein titin in cardiomyocytes was also known to modulate the elasticity of human cardiac tissue and heart disease progression [17]. We previously showed that PDAC development is associated with extensive protein *S*-nitrosylation [18]. Nevertheless, the alterations of protein oxidation profile in pancreatic cancer cells has not yet been investigated previously, and a comprehensive understanding of protein oxidation in pancreatic cancer pathogenesis remains elusive, due to the diversity and dynamic nature of oxidative modifications.

The comprehensive identification of PTM targets is essential for understanding their biological roles. However, the characterization of protein cysteine oxidation the whole-proteome scale has posed a significant technical challenge. This is primarily attributed to the low content of cysteine in proteome, coupled with the diverse forms of oxidative modification on cysteines residues, which arise from the strong electronegativity of sulfur atoms on their side chains [19, 20]. Recently, multiple redox proteomics methodologies have been successfully applied for the efficient characterization of comprehensive protein cysteine oxidation profiles associated with multiple biological processes [9, 12, 19, 21]. Among them, iodoacetyl tandem mass tag (iodoTMT) has been widely used for proteome-wide detection and quantitation of reversibly oxidized cysteine residues owing to its remarkable stability and precision [11, 21]. Recently, the broad applicability of iodoTMT in redox proteomics has been observed across various species and model organisms, including not only mammalian animals but also complex plant tissues [11, 22–25]. However, there remains a scarcity in the application of this proteomic methodology for profiling protein oxidation in pancreatic cancer.

In the present study, we performed a global characterization of protein cysteine oxidation profile associated with PDAC pathogenesis by site-specific quantitative redox proteomics through the combination of iodoTMT and LC–MS/MS methods. Based on proteomic data, the redox states of cysteine residues in hypoxia-inducible factor-1 (HIF-1) signaling proteins were validated in human cancer tissues, further indicating their potential roles in pancreatic cancer pathogenesis. These investigations would provide new insights into molecular events underlying PDAC initiation and progression in term of redox signaling, which could be further explored as new PTM targets for pancreatic cancer early diagnosis and treatment.

## Results

### Global quantification of protein cysteine oxidation in HPDE6c7 and PANC-1 cells

**Fig. 1:**
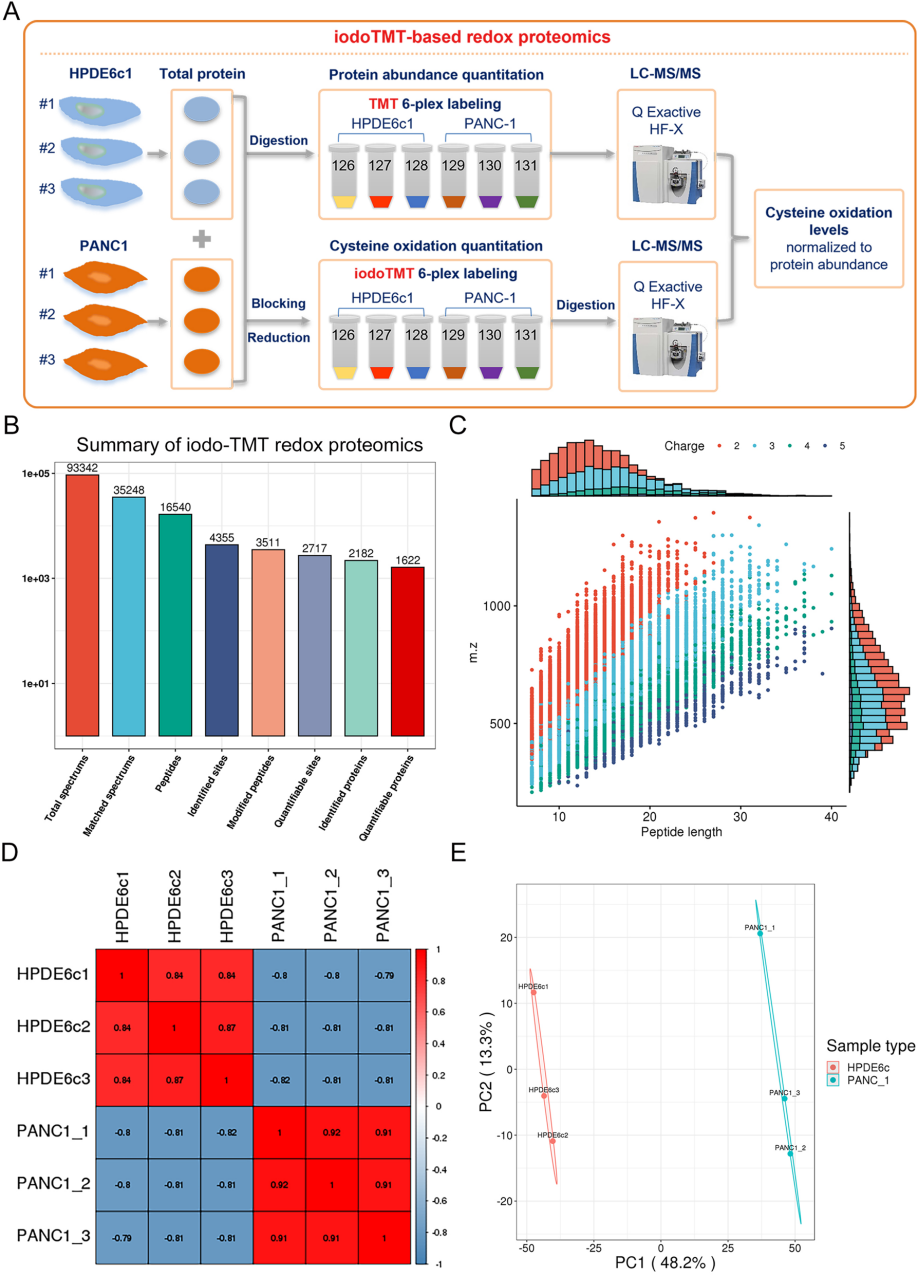
Proteomic characterization of cysteine oxidation in PDAC cell lines. **A** The schematic demonstration of iodoTMT-based redox proteomics used for cysteine oxidation profiling in PDAC cells. Protein oxidation in HPDE6c7 and PANC-1 cells were analyzed by the iodoTMT method using three biological replicates, and cysteine relative oxidation levels were calculated by normalizing to their corresponding expressional abundances measured by TMT quantitative proteomics. **B** A summary of peptides and proteins numbers identified by the iodoTMT-based redox proteomics in HPDE6c7 and PANC-1 cells. **C** The length and charge distribution of oxidized peptides identified in HPDE6c7 and PANC-1 cells. **D** Linear correlations between samples used for iodoTMT proteomics by the Pearson’s Correlation Coefficient. **E** Principal Component Analysis based on the quantitation of protein cysteine oxidation between HPDE6c7 and PANC-1 cell lines. iodoTMT: iodoacetyl tandem mass tag

For a comprehensive understanding of protein oxidation changes during the development of pancreatic cancer, we first carried out a global characterization of oxidized cysteine residues between the HPDE6c7 (immortalized human pancreatic ductal epithelium cell) and PANC-1 cells (PDAC cell line) by the iodoTMT-based quantitative redox proteomic strategy (Fig. 1A). Briefly, oxidized cysteine residues in HPDE6c7 and PANC-1 cells were labelled with different iodoTMT reagents containing distinct isobaric isomers (Thermo Fishier Scientific), followed by LC–MS/MS analysis using a Thermo Q Exactive HF-X mass spectrometer (Fig. 1A). Meanwhile, another round of TMT quantitative proteomics was also performed to compare the global protein abundance differences between these two cell lines (Fig. 1A; Additional file 1: Fig. S1). Finally, the relative cysteine oxidation levels between HPDE6c7 and PANC-1 cells were quantitated by normalizing to their expressional abundances (Fig. 1A). Through three biological replicates, a total of 16,540 peptides were identified in cultured HPDE6c7 and PANC-1 cells, and 4355 oxidized cysteines sites were characterized in totally 2182 modified cysteine-containing proteins (Fig. 1B; Additional file 2: Table S1). Among them, 2717 oxidized cysteine sites were found to be quantifiable, which belongs to totally 1622 quantifiable proteins with oxidative modification (Fig. 1B). Moreover, peptide length distribution showed that the majority of these oxidized cysteine-harboring peptides were composed of 7 to 20 amino acid residues, which is in accordance with the enzymatic digestion and fragmentation methods (Fig. 1C). Furthermore, the repeatability of biological replicates in HPDE6c7 and PANC-1 cell lines were further assessed by the Pearson’s Correlation Coefficient (PCC) and Principal Component Analysis (PCA), which also validated the reliability of this quantitative redox proteomic dataset (Fig. 1D, E). These results collectively demonstrated that this redox proteomic analysis provided a global identification and quantitation of protein cysteine oxidation profiles in pancreatic cancer cells.

### Differential cysteine oxidation profiles between HPDE6c7 and PANC-1 cells

**Fig. 2:**
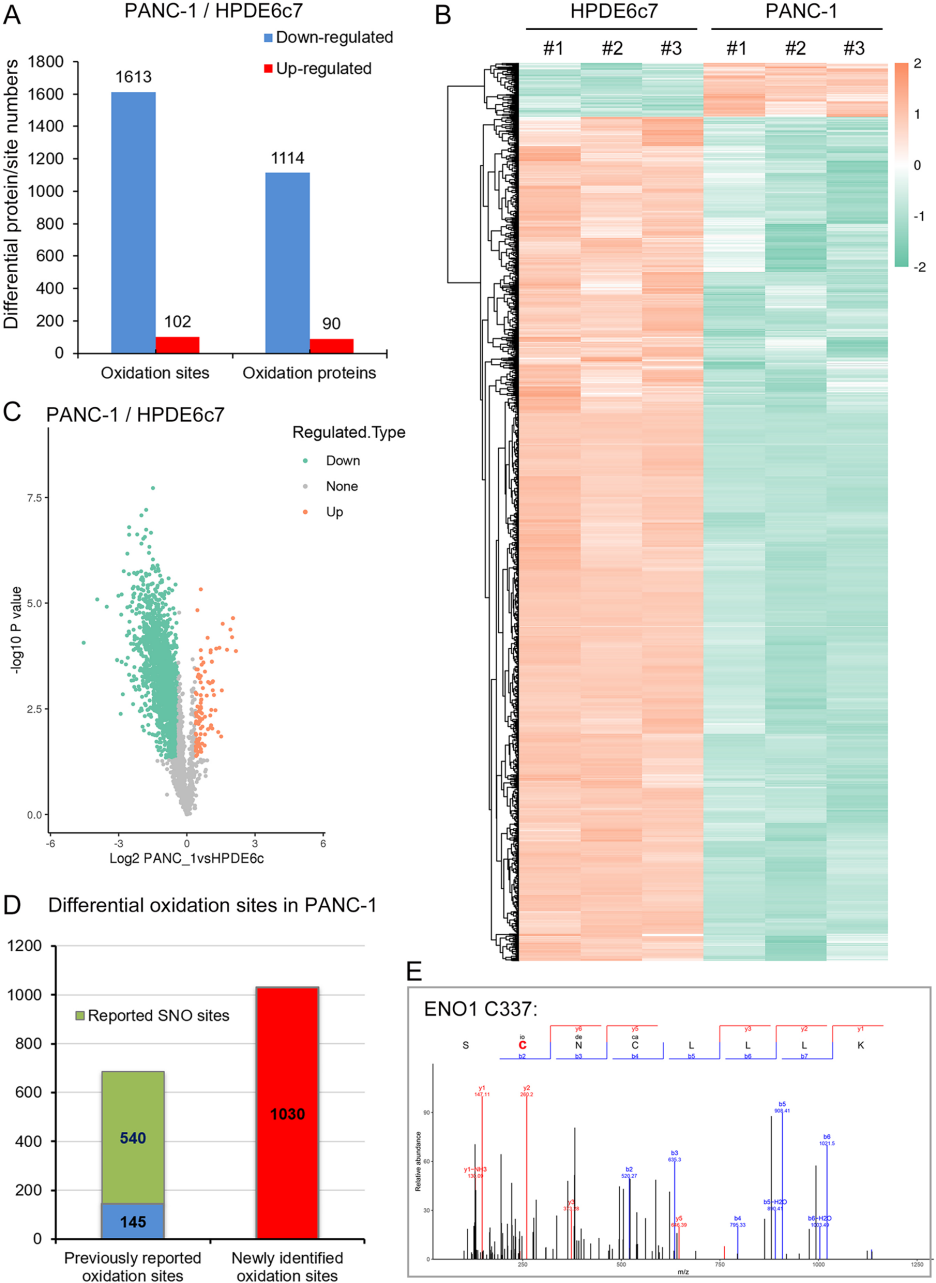
Differential protein cysteine oxidation profiles in PDAC cells. **A** The numbers of cysteine sites and proteins showing differential oxidation levels between HPDE6c7 and PANC-1 cells. **B** The hierarchical clustering of cysteine sites exhibiting differential oxidation between HPDE6c7 and PANC-1 cells. Increased and decreased oxidation levels were indicated by yellow and green colors, respectively. **C** A volcano plot of differentially oxidized cysteine residues between HPDE6c7 and PANC-1 cells. Cysteine sites showing up-regulated and down-regulated oxidation were presented as yellow and green points, respectively. **D** The number of previously reported and newly identified protein cysteine sites characterized in this redox proteomics. Proteins cysteines sites that were identified as S-nitrosylation sites in previous reports were also counted here. **E** The identification of ENO1 protein oxidation at Cys337 in PANC-1 cells by LC–MS/MS method. The oxidized cysteine residue identified by mass spectrometry was shown as red letter. ENO1: enolase-1

To identify protein oxidation targets closely implicated in pancreatic cancer development, we then statistically compared the relative oxidation levels of these identified cysteine residues between HPDE6c7 and PANC-1 cells. Significantly differentially oxidized sites were defined by a P value of < 0.05 and a fold change of > 1.3. In general, our statistical analysis showed that, in total, 1715 protein cysteine residues were differentially oxidized in PANC-1 cells compared with HPDE6c7 cells, including 1613 down-regulated and 102 up-regulated cysteine sites (Fig. 2A; Additional file 3: Table S2). These down-regulated and up-regulated oxidized cysteine sites in PANC-1 cells were matched to 1114 and 90 oxidized proteins respectively (Fig. 2A). These differentially oxidized cysteine sites were then used for hierarchical clustering, which also revealed the significantly different protein cysteine oxidation profiles between HPDE6c7 and PANC-1 cells (Fig. 2B). We also observed that the majority of differentially modified sites exhibited lower oxidation levels in PANC-1 cells compared with the HPDE6c7 cells (Fig. 2B). Similar differential protein oxidation patterns between the HPDE6c7 and PANC-1 cells were also shown by the volcano plot constructed using these differentially oxidized cysteine residues (Fig. 2C). By searching the iCysMod database, we found that 685 differentially oxidized cysteines were previously identified as protein oxidation sites, and among them 540 cysteine sites were also characterized as S-nitrosylation sites in previous reports (Fig. 2D). For instance, the Cys337 in the enolase-1 (ENO1) protein was previously identified as an S-nitrosylation site under multiple contexts [26–28], which was also identified as differential protein cysteine oxidation site between HPDE6c7 and PANC-1 cells in this proteomic study (Fig. 2E). In addition, another set of 1030 differentially oxidized cysteine residues were newly identified as oxidation sites in this study (Fig. 2D). Together, these results suggested significant alterations in protein cysteine oxidation profile of pancreatic cancer cells.

### Consensus sequences of cysteine oxidation in HPDE6c7 and PANC-1 cells

**Fig. 3:**
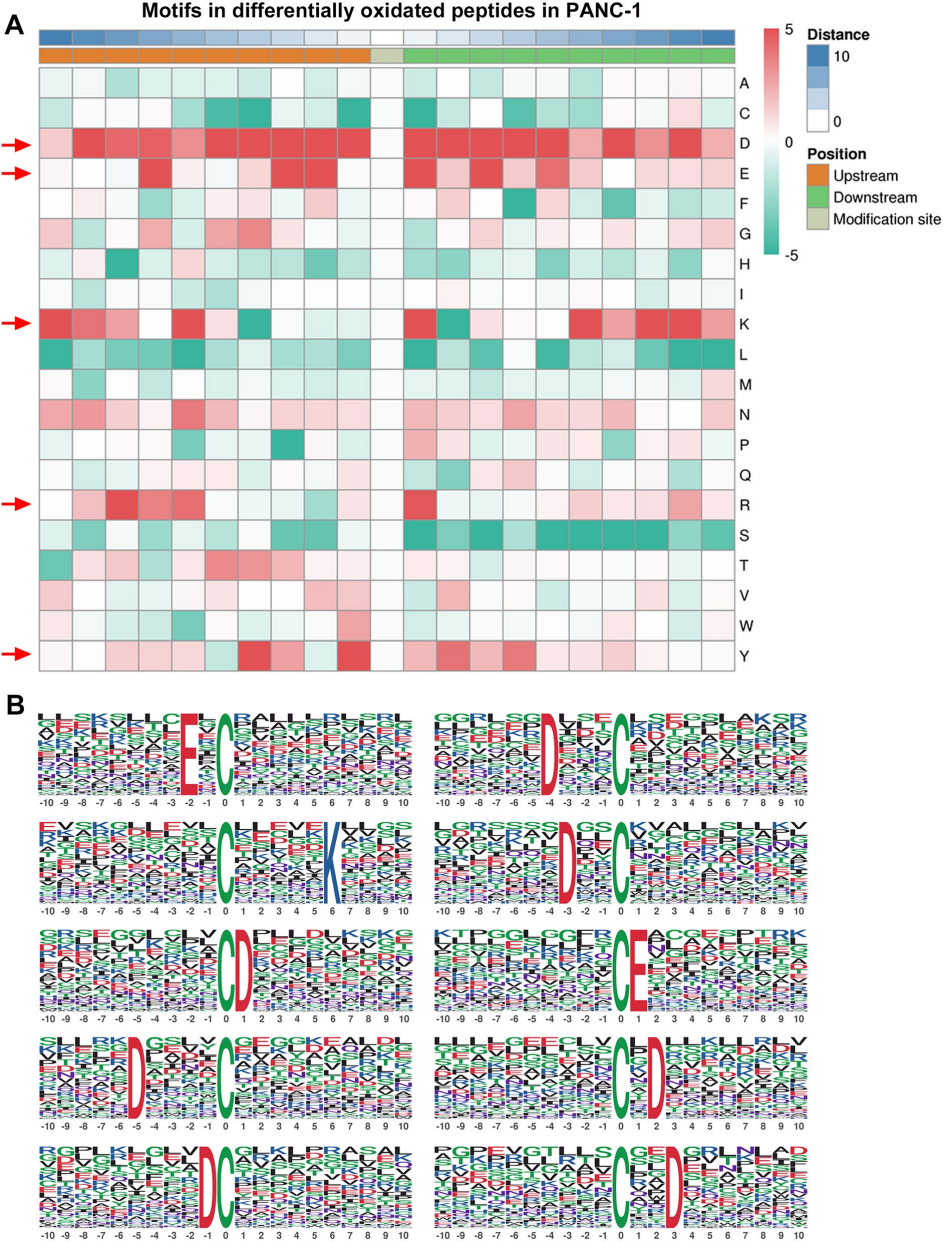
The amino acid composition feature of oxidized peptides in pancreatic cancer cells. **A** The amino acid frequencies of 20 positions flanking cysteine oxidation sites in HPDE6c7 and PANC-1 cells. Animo acid frequencies based on all oxidation peptides identified in this study were analyzed by the MoMo software. High and low amino acid frequencies were indicated by red and green colors, respectively. Significant overrepresentation of amino acids was marked by red arrows. **B** Consensus sequences of protein cysteine oxidation in HPDE6c7 and PANC-1 cells. Statistically significant motifs for protein cysteine oxidation were extracted from all identified oxidation peptides using the Motif-X algorithm

Previous reports demonstrated that the specificity and susceptibility of cysteine residues for S-nitrosylation, one of the major protein oxidation forms, could be substantially influenced by chemical properties of their flanking amino acid composition [18, 29, 30]. However, the specificity of total protein cysteine oxidation is still poorly understood. Here, we also analyzed the features of amino acid composition near oxidized cysteine residues based on all oxidized peptides identified in this study. The frequencies of all 20 common amino acid residues appearing at 20 positions flanking oxidation sites were analyzed using the MoMo software [31], which showed that aspartate (Asp, D), glutamate (Glu, E), lysine (Lys, K), arginine (Arg, R) and tyrosine (Tyr, Y) exhibited significantly higher frequencies at positions in the proximity of oxidized cysteine residues identified in pancreatic cancer cells, compared with other amino acid types (Fig. 3A). Moreover, statistically significant consensus sequences (motifs) were extracted from all oxidation peptides identified in this study via the Motif-X algorithm (Fig. 3B; Additional file 1: Fig. S2). We found that seven protein oxidation motifs from our dataset were featured by an aspartate residue flanking the oxidation site, which is at positions − 5, − 4, − 3, − 1, + 1, + 2 and + 3 respectively (Fig. 3B). Also, glutamate residue was shown to be preferentially present in two other protein oxidation motifs at positions − 2 and + 1 respectively (Fig. 3B). In addition, another oxidation motif containing a lysine residue at position + 6 relative to the modification site was also observed from these oxidized cysteine-harboring peptides identified in HPDE6c7 and PANC-1 cells (Fig. 3B). These results disclosed significant overrepresentations of charged amino acids (both acidic and basic) in residues flanking protein cysteine oxidation sites.

### Functional categorization of differentially oxidized proteins in PANC-1 cells

**Fig. 4:**
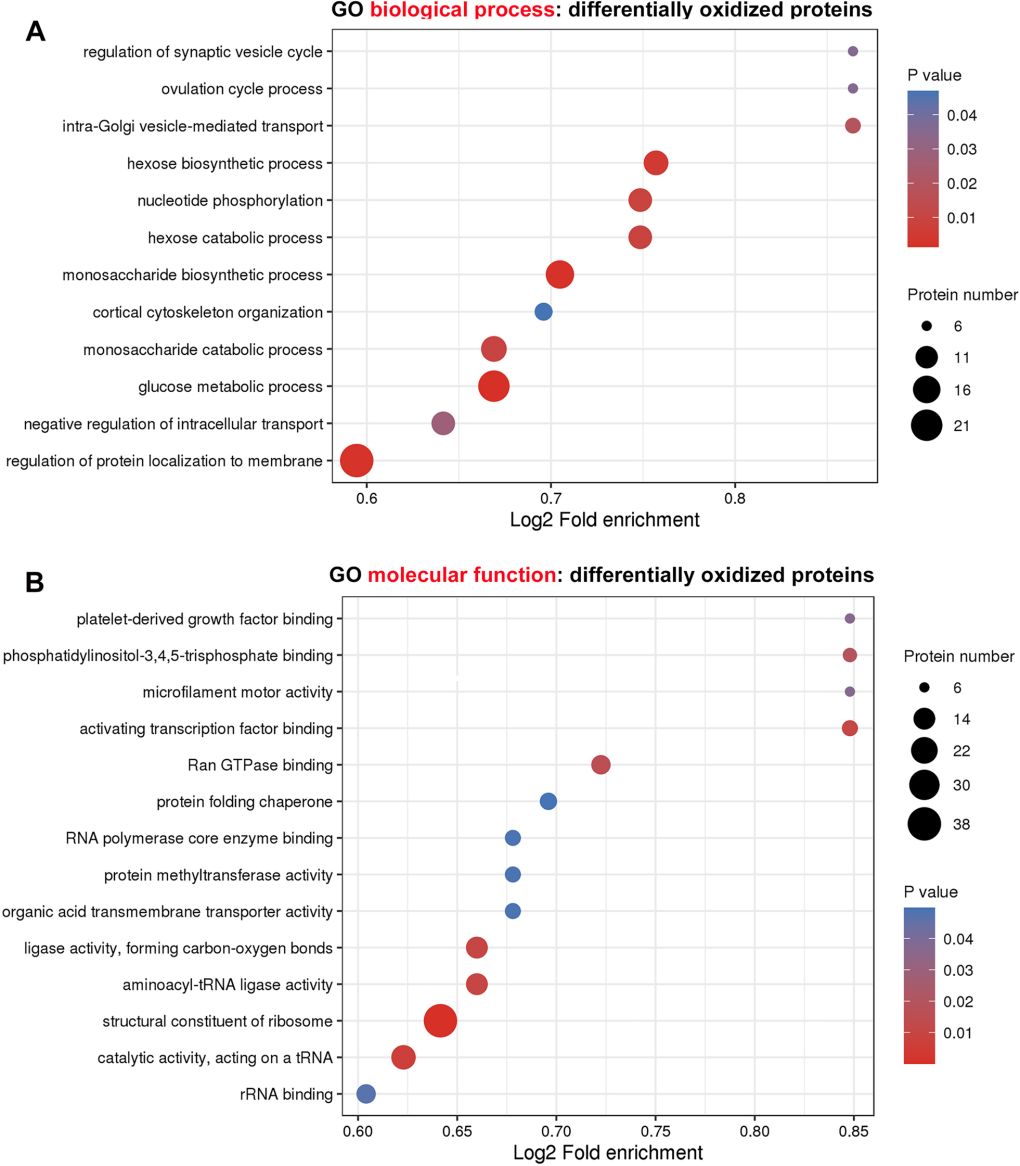
Categorization of proteins differentially oxidized in PDAC by gene ontology. **A** Functional categorization of differentially oxidized proteins in PANC-1 cells based on GO biological processes. **B** The significant enrichment in GO molecular functions of proteins differentially oxidized in PANC-1 cells. The enrichments of biological processes and molecular functions were screened using P < 0.05 as the threshold during analysis with the egg NOG-mapper software. The significance of enrichments and protein numbers were indicated by the color and diameters of circles respectively. GO: gene ontology

For analysis of the potential biological roles of protein oxidation in pancreatic cancer development, we subsequently performed a GO (gene ontology) functional categorization of proteins differentially oxidized between HPDE6c7 and PANC-1 cells using the eggNOG-mapper software. We demonstrated here that these proteins differentially oxidized in PANC-1 cells were significantly enriched in multiple GO biological processes, such as carbohydrate metabolism (glucose metabolism), localization of protein to membranes, intracellular transport regulation, cytoskeleton organization, nucleotide phosphorylation, and intra-Golgi vesicle-mediated transport (Fig. 4A). Moreover, oxidized proteins up-regulated in PANC-1 cells were mainly associated with cytoskeleton organization (extracellular matrix, ECM), cell–cell adhesion, responses to steroid hormone, cell growth and development regulation, transmembrane transporter activity regulation, leukocyte migration, fatty acid metabolism and muscle contraction regulation (Additional file 1: Fig. S3A). Meanwhile, proteins showing down-regulated oxidation in PANC-1 cells were greatly enriched in peptide biosynthesis, localization of proteins to ER, glucose metabolism, ribosome assembly, nucleotide phosphorylation and regulation of cellular response to drugs as well (Additional file 1: Fig. S3B).

Furthermore, differentially oxidized proteins in PANC-1 cells were also significantly enriched in various GO molecular functions, including structural constituent of ribosome, rRNA binding, aminoacyl-tRNA ligase, organic acid transmembrane transporter, protein methyltransferase, RNA polymerase core enzyme binding, protein folding chaperone, Ran GTPase binding, activating transcription factor binding, microfilament motor activity and platelet-derived growth factor binding (Fig. 4B). Similar molecular functions were observed during the categorization of proteins with down-regulated oxidation in PANC-1 cells (Additional file 1: Fig. S4B). However, proteins showing up-regulated oxidation in PANC-1 cells exhibited significantly differential molecular function enrichment, which included cell adhesion molecule binding, integrin binding, calcium ion binding, cholesterol binding, and (insulin-like) growth factor binding as well (Additional file 1: Fig. S4A).

In addition, we also demonstrated based on GO cellular components that differentially oxidized proteins in PANC-1 cells were significantly enriched in multiple subcellular organelles, including cytosolic ribosome, vesicle coat, actin filament, polysome, (histone) methyltransferase complex, complex of collagen trimers, COPI vesicle coat, aminoacyl-tRNA synthetase complex and MLL1/2 complex (Additional file 1: Fig. S5A). Also, our Pfam protein domain analysis displayed that these differentially oxidized proteins in PANC-1 cells have significant enrichment of several protein domains such as Calponin homology (CH) domain, Cyclophilin type peptidyl-prolyl cis–trans isomerase/CLD, Helicase associated domain (HA2) and Myosin head (motor domain) (Additional file 1: Fig. S5B). Together, these observations indicated that protein cysteine oxidation might affect the progression of pancreatic cancer development through modulating various biological processes.

### KEGG pathway enrichment of differentially oxidized proteins in PANC-1 cells

**Fig. 5:**
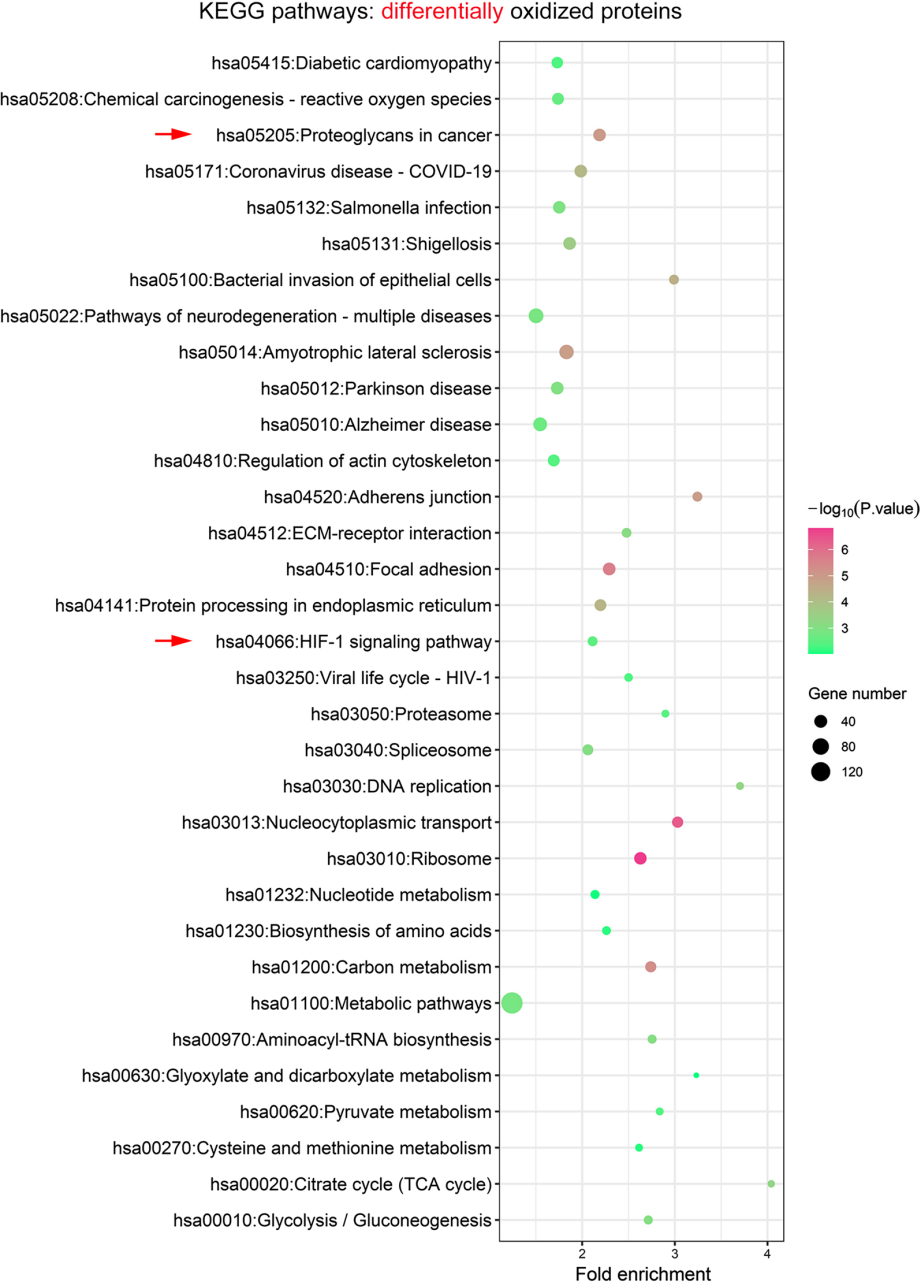
KEGG pathway enrichment of proteins differentially oxidized in PANC-1 cells. All proteins showing differential cysteine oxidation in PANC-1 cells in comparison with the HPDE6c7 cells (Additional file 3: Table S2), were subjected to the KEGG pathway enrichment analysis, through the DAVID website (https://david.ncifcrf.gov/). Each pathway was shown as a circle. The significance of enrichment was evaluated by the P value, which was indicated by the circle colors (High significance: red; Low significance: green). The number of oxidized proteins in each pathway was indicated by circle diameter. KEGG: Kyoto Encyclopedia of Genes and Genomes

For more insights into the pathogenic roles of protein oxidation in PDAC development, we then performed a bioinformatic analysis of KEGG pathways with significant enrichment of proteins differentially oxidized in PANC-1 cells. Specifically, our results showed that these differentially oxidized proteins were also significantly enriched in various KEGG molecular pathways, including multiple metabolic pathways (carbon metabolism, glycolysis/gluconeogenesis, pyruvate metabolism and TCA cycle, aminoacyl-tRNA biosynthesis, cysteine and methionine metabolism, nucleotide metabolism), ribosome, nucleocytoplasmic transport, spliceosome, DNA replication, proteasome, protein processing in endoplasmic reticulum, focal adhesion, ECM-receptor interaction and actin cytoskeleton regulation (Fig. 5). Also, these differentially oxidized proteins were mapped to multiple cancer pathogenic processes including proteoglycans in cancer (Additional file 1: Fig. S7), cell cycle (Additional file 1: Fig. S8), apoptosis (Additional file 1: Fig. S9) and other pathways in cancer (Additional file 1: Fig. S10), as well as other pathogenic processes such as diabetic cardiomyopathy, coronavirus disease (COVID-19), bacterial invasion and even neurogenerative disorders (Fig. 5). More importantly, we also found that differentially oxidized proteins in PANC-1 cells were specifically enriched in one KEGG signaling pathway, the HIF-1 (hypoxia-inducible factor 1) signaling pathway (Fig. 5). Similar enrichment of KEGG pathways was also observed in these proteins showing down-regulated oxidation levels in PANC-1 cells compared with HPDE6c7 (Additional file 1: Fig. S6B). Interestingly, these proteins with up-regulated oxidation in PANC-1 cells was significantly enriched in the PI3K-Akt signaling pathway (Additional file 1: Fig. S6A). Together, these results suggested that protein oxidation might be implicated in pancreatic cancer pathogenesis via multiple physiological and signaling pathways.

### Protein oxidation in HIF-1 signaling pathway associated with PDAC development

**Fig. 6:**
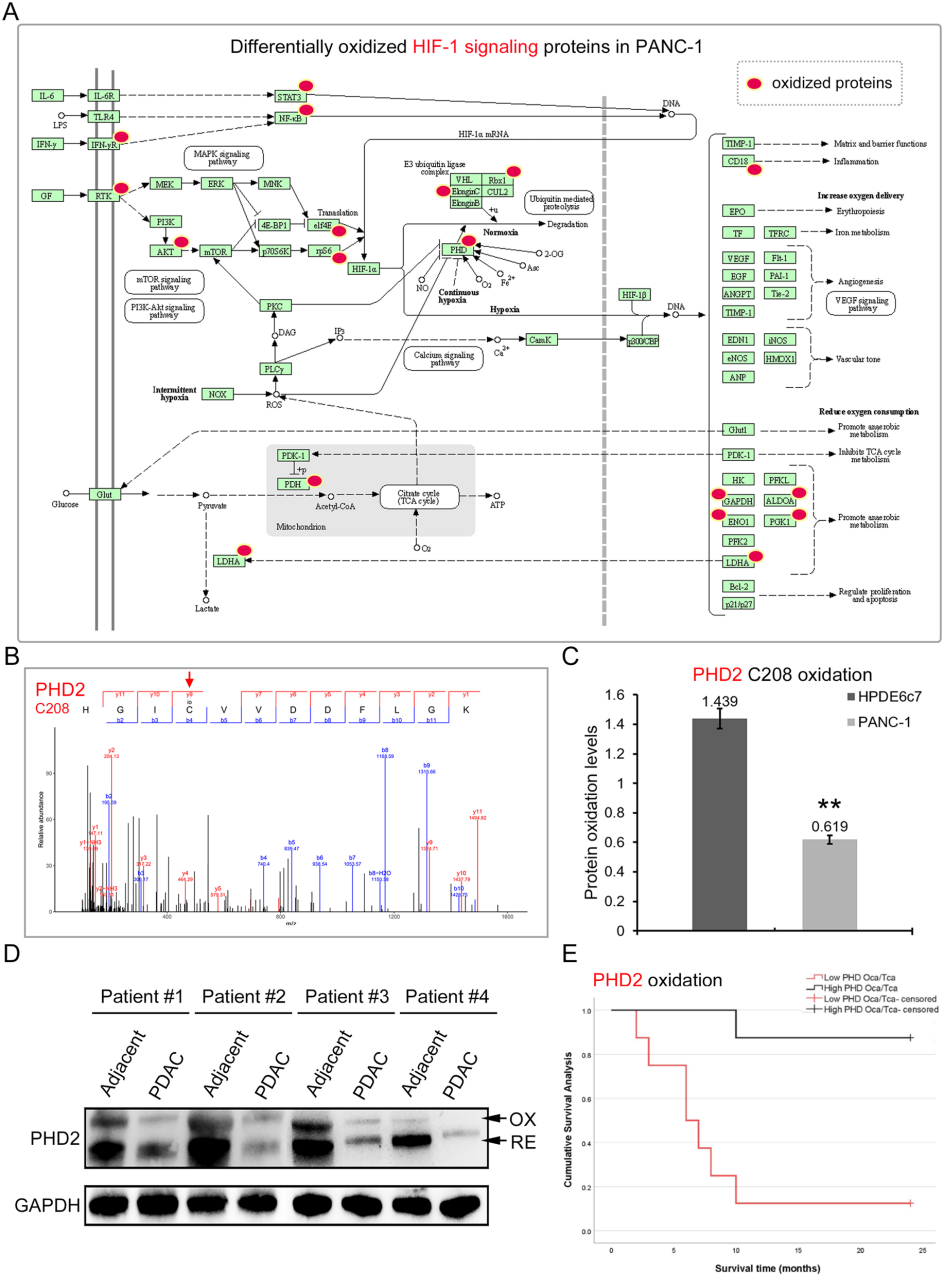
Modulation of HIF-1 signaling by cysteine oxidation in PDAC. **A** Differential cysteine oxidation of HIF-1 signaling proteins in PANC-1 cells in contrast to the HPDE6c7 cells. HIF-1 signaling proteins with significant alteration of cysteine oxidation levels between the HPDE6c7 and PANC-1 cells were marked by red ovals. Detailed information of their quantitation could be found in Additional file 3: Table S2. The signaling route Fig. was modified from the KEGG database. **B**, **C** Decreased oxidation of PHD2 Cys208 site in PANC-1 cells. The oxidation of Cys208 in PHD2 protein was identified by LC–MS/MS (**B**) and quantitated by label-free strategy (**C**). **D** Alteration of PHD2 protein oxidation in cancerous tissues from PDAC patients. Total proteins from cancerous and adjacent tissues were subjected to AMS labeling followed by western blotting against PHD. OX: oxidized; RE: reduced. **E** The association of PHD protein oxidation and survival times in human PDAC patients. PHD Oca/Tca indicates the ratio of oxidized PHD2 protein levels to total PHD2 protein abundances in pancreatic cancer tissues. Totally 16 PDAC patients were enrolled in this study and used for the survival time analysis. PHD2: prolyl hydroxylase domain 2; **P < 0.01

Based on our redox proteomics dataset, the HIF-1 signaling pathway underwent substantial protein cysteine oxidation in PDAC cell line. Specifically, a total of 17 signaling protein components of the HIF-1 pathway was shown to be differentially oxidized in PANC-1 cells compared with the HPDE6c7 cells, including STAT3 (Signal Transducer And Activator Of Transcription 3), NF-κB (nuclear factor kappa-B), IFN-γR (Interferon-γ receptor), AKT (Protein kinase B), RTK (Receptor tyrosine kinase), eIF4E (Eukaryotic translation initiation factor 4E), RPS6 (Ribosomal Protein S6), PHD2 (prolyl hydroxylase domain 2), RBX1 (RING box protein 1), Elongin-C, PDH (pyruvate dehydrogenase), LDHA (Lactate Dehydrogenase A), CD18, GAPDH (Glyceraldehyde-3-Phosphate Dehydrogenase), ALDOA (Aldolase, Fructose-Bisphosphate A), ENO1 (Enolase 1) and PGK1 (Phosphoglycerate Kinase 1) (Fig. 6A; Additional file 3: Table S2). For instance, the Cys208 of PHD2 protein was identified here as one oxidation site in PANC-1 cells by LC–MS/MS method (Fig. 6B). Moreover, quantitation analysis based on TMT proteomics showed that, compared with the HPDE6c7 cells, the oxidation level of PHD2 Cys208 site was greatly reduced in PANC-1 cells (Fig. 6C). In addition, the Cys251 residue of STAT3 protein was also characterized as cysteine oxidation site in this proteomic assay (Additional file 1: Fig. S11A). Similarly, the oxidation level of STAT3 Cys251 site in PANC-1 cells was significantly lower than that in the HPDE6c7 cells (Additional file 1: Fig. S11B).

To further validate the implication of HIF-1 signaling protein oxidation in PDAC pathogenesis, we then confirmed the alteration of PHD2 protein oxidation levels in human cancer tissues. The relative levels of oxidized and reduced PHD2 levels in cancerous and corresponding adjacent non-cancerous tissues from 16 PDAC patients were determined by AMS labeling and western blotting as described in the Method section (Fig. 6D). In consistence with the above-mentioned proteomic results, we found that the oxidized PHD2 protein levels in PDAC cancerous tissues were significantly reduced compared to their corresponding adjacent non-cancerous tissues (Fig. 6D). Furthermore, KM survival analysis shows that PHD2 protein oxidation in pancreatic cancer tissue is closely related to the survival time of pancreatic cancer patients, and the survival time of patients with lower levels of PHD2 protein oxidation is significantly lower than those with higher PHD2 protein oxidation (8.25 months VS 22.25 months, P = 0.001) (Fig. 6E). Together, these results indicated that the modulation of HIF-1 signaling pathway by protein oxidation might play pivotal roles in pancreatic cancer development and progression.

## Discussion

Protein cysteine oxidation, a set of post-translational modifications prevalently occurring in various organisms [10, 11, 32], has been extensively recognized as one functionally critical mechanism driving human disease pathogenesis, such as diabetes, atherosclerosis, ischemia reperfusion, cancers and neurodegeneration [10, 33–36]. However, the roles of cysteine oxidation in pancreatic cancer development still remains poorly elucidated. In the present study, we performed the global characterization of oxidized cysteine-containing proteins in PDAC cell line via iodoTMT-based quantitative redox proteomics, which demonstrated the significant alterations of protein cysteine oxidation profiles in pancreatic cancer cells, in contrast to normal pancreatic cells. Our following bioinformatic analyses showed that multiple biological processes and signaling pathways underwent extensive oxidative cysteine modifications in pancreatic cancer cells including the HIF-1 signaling pathway. The oxidation status of PHD2 protein, one major signaling protein in HIF-1 pathway, was demonstrated here to be remarkably changed in both PDAC cell line and human cancerous tissues, which was significantly correlated with lower survival time in PDAC patients. These investigations clearly showed for the first time the great alterations of total protein cysteine oxidation profiles under the context of pancreatic cancer.

In order to provide accurate quantitation of oxidative modification, the relative cysteine oxidation levels of individual proteins between HPDE6c7 and PANC-1 cells were calculated by normalizing to their corresponding protein abundances in these two cell lines. Based on this label-free quantitative method, a large set of functional proteins were shown to be significantly differentially oxidized in PANC-1 cells compared with the normal pancreatic cell line HPDE6c7. Interestingly, a large number of down-regulated oxidation peptides were characterized in PANC-1 cells, much more than those showing up-regulated oxidation levels (Fig. 1), although similar numbers of proteins showing increased and decreased abundances were observed in PANC-1 cells during TMT quantitation (Additional file 1: Fig. S1). These observations suggested that the modulation of protein functions by cysteine oxidation might also play dual roles in pancreatic cancer development depending on the disease stage and progression speed. Our previous research demonstrated extensive protein S-nitrosylation that modulated pleiotropic biological processes and signaling pathways during PDAC development [18]. In this study, we also observed that 685 differentially oxidized sites between HPDE6c7 and PANC-1 cells were previously identified as oxidized cysteines, including 540 S-nitrosylation sites. Meanwhile, over 1000 cysteine sites were newly characterized as oxidation targets in this study, showing the specific features of protein oxidation profiles associated with pancreatic physiology and PDAC pathogenesis. In addition, we found that the flanking sequences of oxidation sites here were featured by the overrepresentation of acidic and basic amino acid residues, which is also consistent with previous reports supporting the acid-basic motif in facilitating protein S-nitrosylation [18, 29, 30, 37]. It should be noted that the variation of protein oxidation disclosed here in cultured cancer cells needs further validation due to the potential differences between culture environment and in vivo conditions.

Our subsequent bioinformatic analysis demonstrated these differentially oxidized proteins in HPDE6c7 and PANC-1 cells were significantly enriched in various biological processes including multiple metabolism and transport processes. Moreover, significantly different biological process enrichments were observed between up-regulated and down-regulated oxidized proteins in PANC-1 cells, suggesting the multi-faceted roles of protein oxidation in regulating pancreatic cancer initiation and progression. The complexity of protein oxidation function in PDAC was also supported by their enrichments in various molecular functions and subcellular components. For instance, protein methylation catalyzed by PRMTs (protein arginine methyltransferases) and other methyltransferases has been recently established as one key molecular event regulating pancreatic carcinogenesis and treatment [38, 39]. Our previous proteomic research also demonstrated PDAC cells exhibit great changes of PRMT-mediated protein methylation, which might act as one essential molecular pathogenic mechanism underlying pancreatic cancer development [40]. In addition, we also showed that protein S-nitrosylation, the cysteine oxidation form induced by nitric oxide (NO), can regulate protein arginine methylation during redox signaling and stress responses [41]. Interestingly, we found in this study that differentially oxidized proteins were significantly enriched in protein methyltransferase activity and histone methyltransferase complex (Additional file 1: Figs. S4B and S5A). Together, these results suggest that the interplay between protein oxidation and other PTM types such as protein methylation might also play essential roles in PDAC development and progression.

Importantly, we disclosed that proteins differentially oxidized in PDAC cells were significantly enriched in the HIF-1 signaling pathway. Hypoxia-inducible factor 1a (HIF-1a), known as the master modulator of oxygen homeostasis, functions as a key transcription factor regulating the expression of genes involved in various aspects of cellular processes and cancer biology [42, 43]. The development and metastasis of pancreatic cancer could also be promoted by hypoxia-induced HIF-1a overexpression, mediated by the expressional activation of various functional genes [44]. On the other hand, the stability of HIF-1a protein could be negatively regulated by prolyl hydroxylases (PHDs), which hydroxylate HIF-1a protein to promotes the proteasome-mediated HIF-1a degradation [43]. However, the mechanisms of PHD regulation in context of pancreatic cancer are still poorly understood. In this study, we showed that the oxidation of PHD2 protein, also termed as EGLN1 (Egl nine homolog 1), in PDAC cells was significantly lowered in PDAC cells. The reduced PHD2 protein oxidation was further confirmed in human pancreatic cancer tissues, which exhibited significant correlation with survival time of PDAC patients. In contrast, we also showed that HIF-1a protein oxidation was greatly elevated in human pancreatic cancer tissues, which was positively correlated with PDAC progression and poor prognosis. Based on these observations, it could be proposed that lowered PHD2 oxidation might impair its catalytic activity, thus leading to the elevation of HIF-1a protein abundance and the enhancement of target gene expression to promote pancreatic cancer development. Of note, this research is limited by the lack of in vivo validation using more physiologically relevant models, which is crucial for enhancing the translational potential of the above findings.

In summary, we reported here the first global profiling of differentially oxidized proteins in pancreatic cancer cells via iodoTMT-based quantitative redox proteomics, which were functionally enriched in various biological processes and signaling pathways. Importantly, our further assays demonstrated that the HIF-1 signaling pathway underwent significant oxidation changes in pancreatic cancer cells, and the altered oxidation in PHD2 proteins was closely correlated with survival time in PDAC patients. These investigations provided novel insights into the redox-mediated molecular mechanisms underlying PDAC pathogenesis, which might also facilitate the development of new targets for pancreatic cancer diagnosis and treatment.

## Materials and methods

### Cell culture and experimental design

The immortalized human pancreatic ductal epithelium cell line HPDE6c7 was obtained from the Kyushu University (Japan) and PDAC cell line PANC-1 was purchased from the Type Culture Collection of the Chinese Academy of Sciences (Shanghai, China). Short tandem repeat (STR) profiling was used to authenticate cell identity. Cells were cultured in DMEM (Dulbecco's Modified Eagle Medium) containing 10% fetal bovine serum (Invitrogen) and penicillin/streptomycin at 37^◦^C with 5% CO2. HPDE6c7 and PANC-1 cells cultured under the same conditions as three biological replicates (n = 3), were analyzed by iodoTMT redox proteomics for discovery of oxidized proteins (details in Fig. 1A). Reliable identification of oxidized peptides was finally obtained by searching with MaxQuant (1.6.0.16) using a FDR of < 1% at both the peptide and protein group levels for control of false identification.

### Protein extraction

Cell sample was sonicated three times on ice using a high intensity ultrasonic processor (Scientz) in lysis buffer (8 M urea, 1% protease inhibitor cocktail). (For PTM experiments, inhibitors were also added to the lysis buffer, e.g. 3 μM TSA and 50 mM NAM for acetylation, 1% phosphatase inhibitor for phosphorylation). The remaining debris was removed by centrifugation at 12,000 g at 4 °C for 10 min. Finally, the supernatant was collected and the protein concentration was determined with BCA kit according to the manufacturer’s instructions.

### Trypsin digestion and labeling

For digestion, the protein solution was reduced with 5 mM dithiothreitol for 30 min at 56 °C and alkylated with 11 mM iodoacetamide for 15 min at room temperature in darkness. The protein sample was then diluted by adding 100 mM TEAB to urea concentration less than 2 M. Finally, trypsin was added at 1:50 trypsin-to-protein mass ratio for the first digestion overnight and 1:100 trypsin-to-protein mass ratio for a second 4 h-digestion. Finally, the peptides were desalted by C18 SPE column. Tryptic peptides were firstly dissolved in 0.5 M TEAB. Each channel of peptide was labeled with its respective TMT reagent (based on manufacturer’s protocol, Thermo Fisher Scientific), and incubated for 2 h at room temperature. Five microliters of each sample were pooled, desalted, and analyzed by MS to check labeling efficiency. After checking labeling efficiency, samples were quenched by adding 5% hydroxylamine. The pooled samples were then desalted with a Strata X C18 SPE column (Phenomenex) and dried by vacuum centrifugation.

### HPLC fractionation

The sample was fractionated into fractions by high pH reverse-phase HPLC using an Agilent 300 Extend C18 column (5 μm particles, 4.6 mm ID, 250 mm length). Briefly, peptides were separated with a gradient of 2 to 60% acetonitrile in 10 mM ammonium bicarbonate pH 10 over 80 min into 80 fractions. Then, the peptides were combined into 9 fractions and dried by vacuum centrifugation.

### LC–MS/MS analysis

The tryptic peptides were dissolved in solvent A (0.1% formic acid, 2% acetonitrile in water), directly loaded onto a home-made reversed-phase analytical column (25-cm length, 75 μm i.d.). Peptides were separated with a gradient from 5 to 25% solvent B (0.1% formic acid in 90% acetonitrile) over 60 min, 25 to 35% in 22 min and climbing to 80% in 4 min then holding at 80% for the last 4 min, all at a constant flow rate of 450 nL/min on an EASY-nLC 1200 UPLC system (Thermo Fisher Scientific). The separated peptides were analyzed in a Q Exactive™ HF-X (Thermo Fisher Scientific) with a nano-electrospray ion source. The electrospray voltage applied was 2.0 kV. The full MS scan resolution was set to 60,000 for a scan range of 350–1600 m/z. Up to 20 most abundant precursors were then selected for further MS/MS analyses with 30 s dynamic exclusion. HCD fragmentation was performed at a normalized collision energy (NCE) of 28%. The fragments were detected in the Orbitrap at a resolution of 30,000. Fixed first mass was set as 100 m/z. Automatic gain control (AGC) target was set at 1E5, with an intensity threshold of 3.3E4 and a maximum injection time of 60 ms.

### Database search

The resulting MS/MS data were processed using MaxQuant search engine (v.1.6.15.0). Tandem mass spectra were searched against the human SwissProt database (20,422 entries) concatenated with reverse decoy database. Trypsin/P was specified as cleavage enzyme allowing up to 2 missing cleavages. The mass tolerance for precursor ions was set as 20 ppm in first search and 5 ppm in main search, and the mass tolerance for fragment ions was set as 0.02 Da. Carbamidomethyl on Cys was specified as fixed modification, and acetylation on protein N-terminal and oxidation on Met were specified as variable modifications. FDR was adjusted to < 1%. Raw data and search result files were submitted to the ProteomeXchange Consortium (http://www.proteomexchange.org/) via the iProX partner repository, with the dataset identifier PXD043973.

### Quantitation and bioinformatic analysis

Sample repeatability of protein oxidation quantitation in this study was evaluated through the Principal Component Analysis and the Pearson’s Correlation Coefficient analysis. For characterizing differentially oxidized peptides, their reporter intensities from LC–MS/MS analysis were first subjected to centralization transformation, followed by normalizing to their corresponding protein expressional abundances. Then, relative oxidation levels of identified cysteine sites between HPDE6c7 and PANC-1 cell lines were assessed by the Student’s t-test, and significantly differential oxidation was defined by a fold change of 1.3 and a P value of < 0.05. The amino acid composition feature was analyzed using both the MoMo software (meme-suite.org) and Motif-X algorithm (motif-x.med.harvard.edu). Functional categorization of differentially oxidized proteins between these two cell lines was performed based on GO biological processes, molecular functions and cellular components respectively, using a P value of < 0.05 in Fisher’s exact test as the threshold for significant enrichments. Meanwhile, the enrichment of differentially oxidized proteins between HPDE6c7 and PANC-1 cells on KEGG (Kyoto Encyclopedia of Genes and Genomes) pathways were analyzed using the DAVID platform (https://david.ncifcrf.gov/), and significant enrichment was defined a P value of < 0.05.

### Oxidation validation in PDAC tissues

Totally 16 PDAC patients that underwent surgical treatment in Hunan Provincial People's Hospital between 2021 and 2022 were enrolled for this study, and all patients were followed up for over 24 months. Their PDAC tissues and corresponding non-cancerous adjacent pancreatic tissues were collected during surgery and subjected to the determination of oxidized and reduced PHD2 protein levels through western blotting following AMS labelling, as previous introduced with minor modifications [22]. AMS (4-acetamido-4'-maleimidylstilbene-2,2'-disulfonic acid, disodium salt) is a thiol-reactive fluorescent probe that specifically labels the oxidized form of protein thiols, thus causing the separation of oxidized and reduced forms of one protein during SDS-PAGE analysis [45]. Briefly, pancreatic tissues were homogenized in tissue lysis buffer for extraction of total proteins. Reduced proteins were labeled by incubation with 4-acetamido-4'-maleimidylstilbene-2,2'-disulfonic acid, disodium salt (AMS) (8 mM) for 1 h at room temperature in darkness. Subsequently, the relative abundances of oxidized or reduced PHD2 proteins were measured by standard western blotting procedures. For survival analysis, pancreatic cancer patients were divided into high and low PHD2 oxidation groups, and the difference of survival time between these two groups was compared.

### Supplementary Information

**Additional file 1: Fig. S1.** Protein abundance differences between HPDE6c7 and PANC-1 cells revealed by TMT proteomics. **Fig. S2.** Details of cystine oxidation motifs identified in PANC-1 cells. **Fig. S3.** GO biological processes with significant enrichment of oxidized proteins up-regulated or down-regulated in PANC-1 cells. **Fig. S4.** GO molecular functions with significant enrichment of oxidized proteins up-regulated or down-regulated in PANC-1 cells. **Fig. S5.** GO cellular components with significant enrichment of oxidized proteins up-regulated or down-regulated in PANC-1 cells. **Fig. S6.** KEGG pathways with significant enrichment of oxidized proteins up-regulated or down-regulated in PANC-1 cells. **Fig. S7.** Oxidized proteins in the proteoglycans in cancer pathway. **Fig. S8.** Oxidized proteins in the cell cycle pathway. **Fig. S9.** Oxidized proteins in the apoptosis pathway. **Fig. S10.** Oxidized proteins in the cancer-related pathways. **Fig. S11.** Decreased STAT3 oxidation in PANC-1 cells.

**Additional file 2: Table S1.** Detailed information of protein oxidization sites identified in HPDE6c7 and PANC-1 cells.

**Additional file 3: Table S2.** Differentially oxidized peptides in PANC-1 cell line compared with HPDE6c7.

## Data Availability

All datasets used to support the findings of this research are available in this article or in the Additional files. Meanwhile, the raw data and search result files from LS-MS/MS experiments were submitted to the ProteomeXchange Consortium (http://www.proteomexchange.org/; dataset identifier: PXD043973).

## Abbreviations

AD: Alzheimer’s disease
ECM: Extracellular matrix
ENO1: Enolase-1
GO: Gene Ontology
HIF-1: Hypoxia-inducible factor-1
iodoTMT: Iodoacetyl tandem mass tag
IRE1α: Inositol-requiring protein 1α
KEGG: Kyoto Encyclopedia of Genes and Genomes
EGLN1: Egl nine homolog 1
NF-κB: Nuclear factor kappa-B
PCA: Principal component analysis
PCC: Pearson’s correlation coefficient
PDAC: Pancreatic ductal adenocarcinoma
PDH: Pyruvate dehydrogenase
PGK1: Phosphoglycerate kinase 1
PHD2: Prolyl hydroxylase domain 2
PRMTs: Protein arginine methyltransferases
PTM: Protein translational modification
ROS: Reactive oxygen species
RTK: Receptor tyrosine kinase
SNO: S-nitrosylation
STAT3: Signal transducer and activator of transcription 3
TBI: Traumatic brain injury
UPR: Unfolded protein response
