# Supplementary material for: Redox proteomics of PANC-1 cells reveals the significance of HIF-1 signaling protein oxidation in pancreatic ductal adenocarcinoma pathogenesis
Source: J Transl Med. 2024 Mar 16;22:287. doi: 10.1186/s12967-024-05068-z (PMC10944602; doi:10.1186/s12967-024-05068-z)
Supplement: Supplementary file 1 — Additional file 1: Fig. S1. Protein abundance differences between HPDE6c7 and PANC-1 cells revealed by TMT proteomics. Fig. S2. Details of cystine oxidation motifs identified in PANC-1 cells. Fig. S3. GO biological processes with significant enrichment of oxidized proteins up-regulated or down-regulated in PANC-1 cells. Fig. S4. GO molecular functions with significant enrichment of oxidized proteins up-regulated or down-regulated in PANC-1 cells. Fig. S5. GO cellular components with significant enrichment of oxidized proteins up-regulated or down-regulated in PANC-1 cells. Fig. S6. KEGG pathways with significant enrichment of oxidized proteins up-regulated or down-regulated in PANC-1 cells. Fig. S7. Oxidized proteins in the proteoglycans in cancer pathway. Fig. S8. Oxidized proteins in the cell cycle pathway. Fig. S9. Oxidized proteins in the apoptosis pathway. Fig. S10. Oxidized proteins in the cancer-related pathways. Fig. S11. Decreased STAT3 oxidation in PANC-1 cells. [file 12967_2024_5068_MOESM1_ESM.docx]

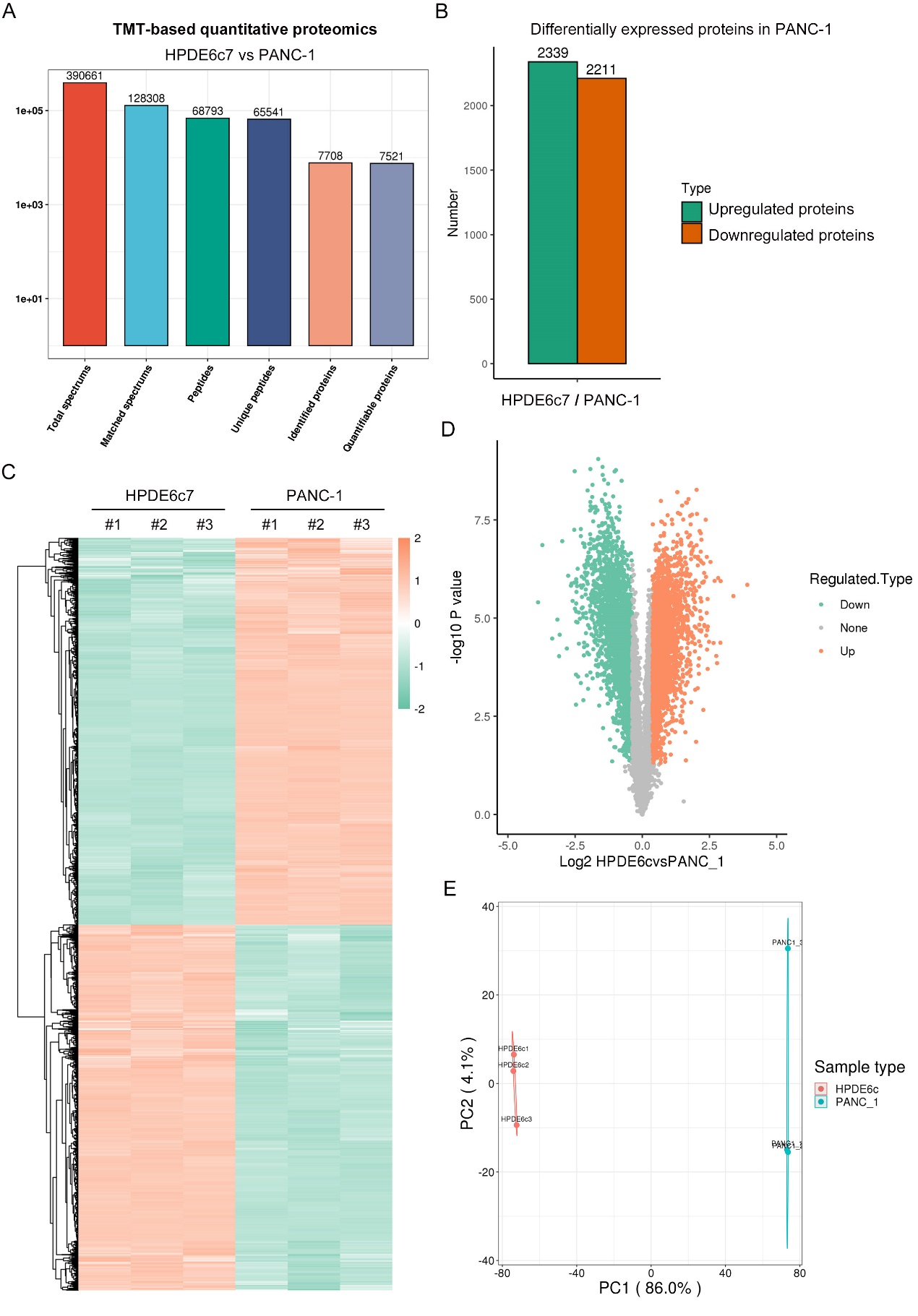


**Fig. S1. Protein abundance differences between HPDE6c7 and PANC-1 cells revealed by TMT proteomics.**

The total protein expressional differences between HPDE6c7 and PANC-1 cells were evaluated by TMT quantitation proteomics, which was used for calibration of protein oxidization changes. (A) Summary of TMT proteomics; (B) Number of differentially expressed proteins; (C) The hierarchical clustering of differentially expressed proteins; (D) A volcano plot of differentially expressed proteins. (E) Principal component analysis of differentially expressed proteins.


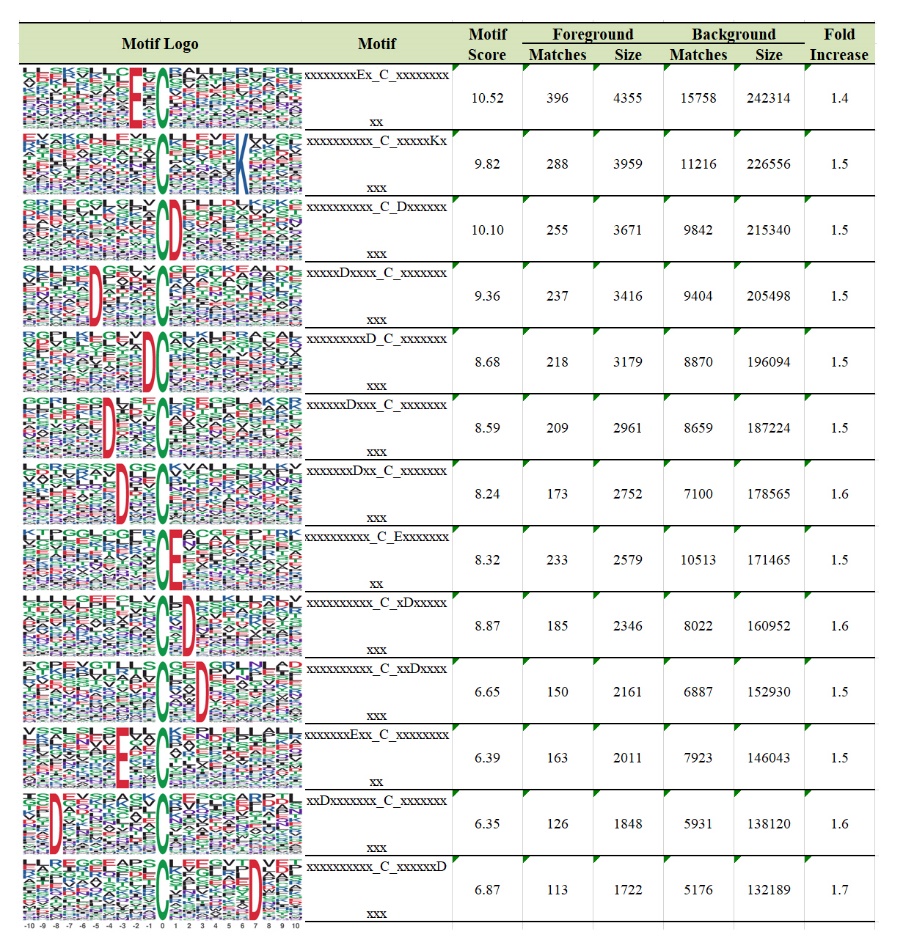


**Fig. S2. Details of cystine oxidation motifs identified in PANC-1 cells.**

Statistically significant consensus sequences (motifs) were extracted from all oxidized peptides using the Motif-X algorithm.

**
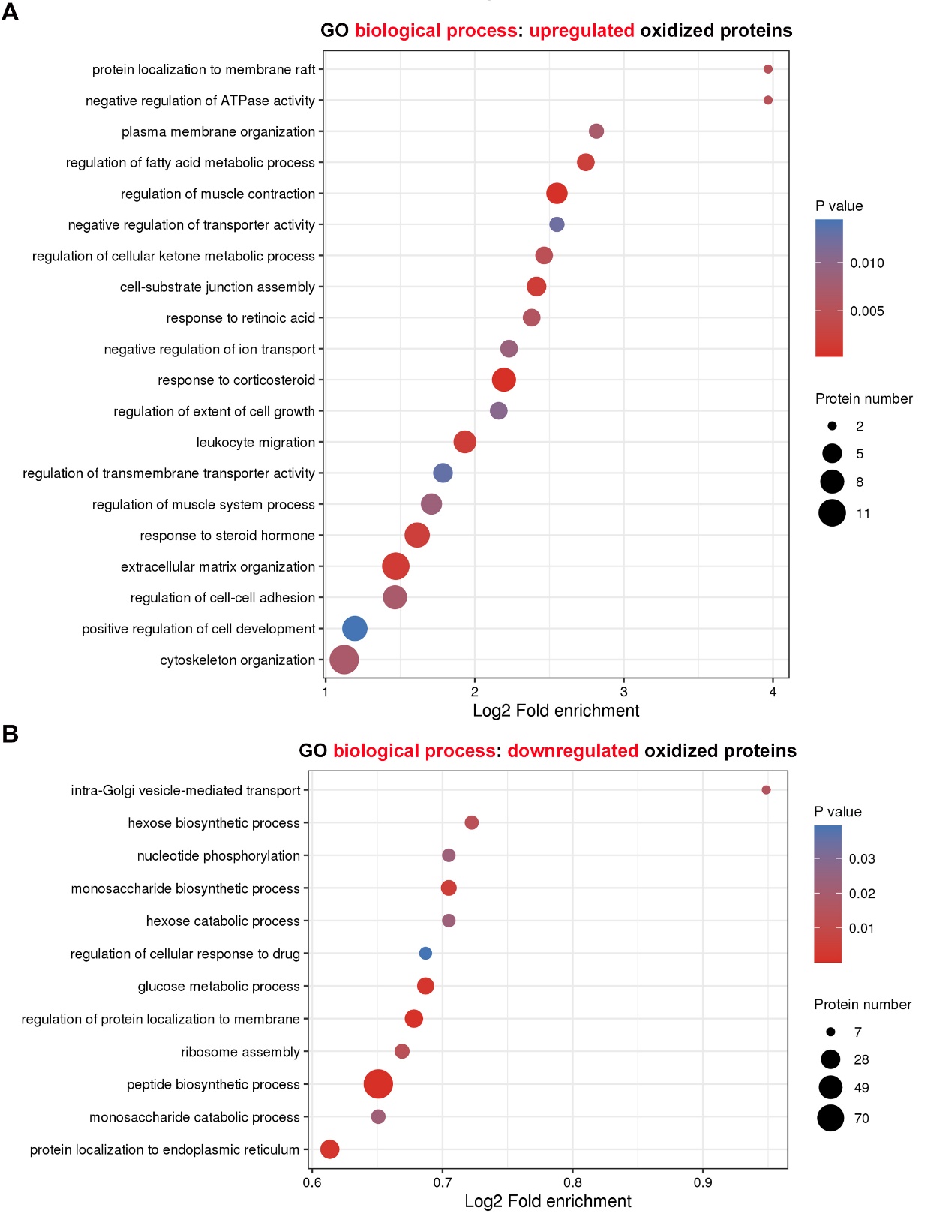
**

**Fig. S3. GO biological processes with significant enrichment of oxidized proteins up-regulated or down-regulated in PANC-1 cells.**

**
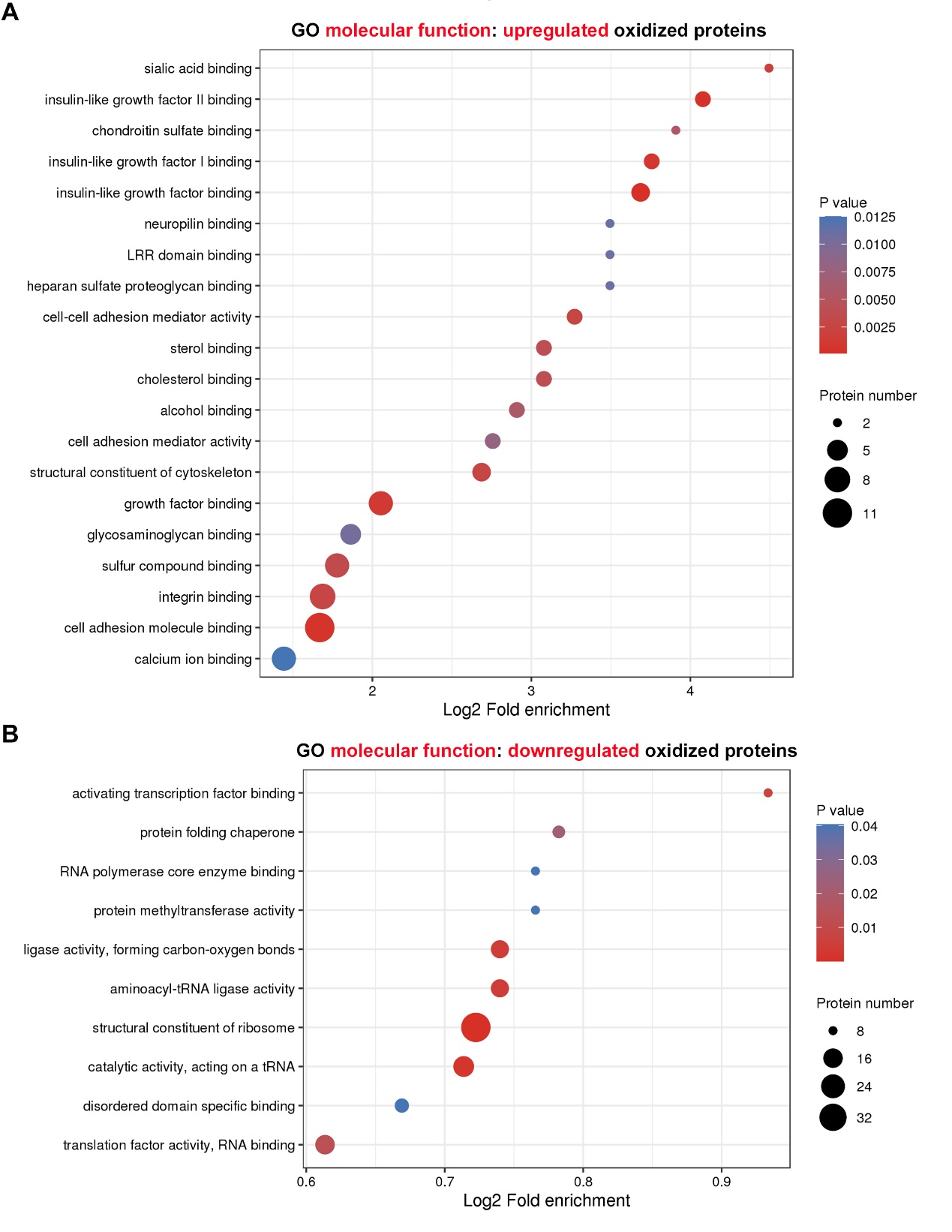
**

**Fig. S4. GO molecular functions with significant enrichment of oxidized proteins up-regulated or down-regulated in PANC-1 cells.**

**
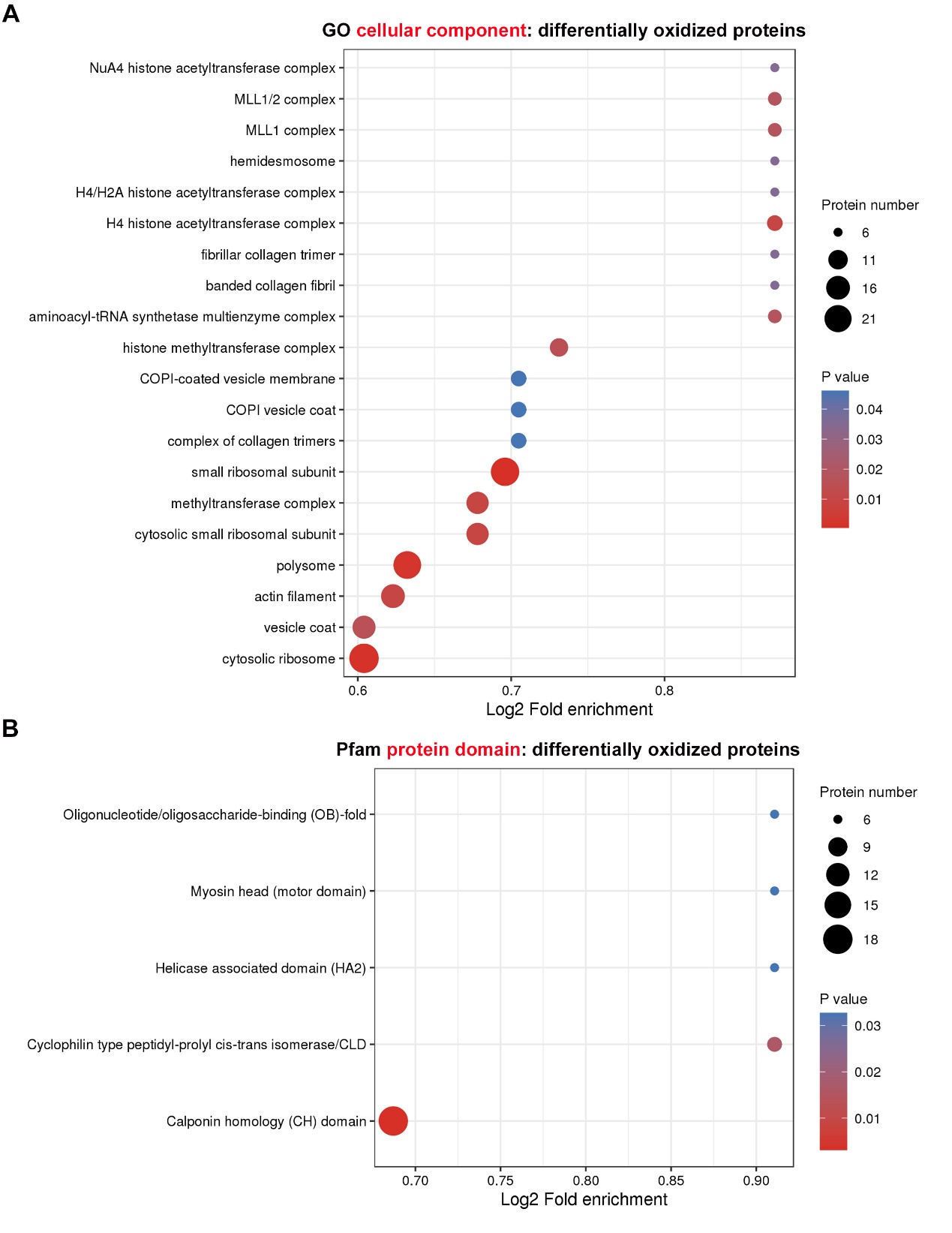
**

**Fig. S5. GO cellular components with significant enrichment of oxidized proteins up-regulated or down-regulated in PANC-1 cells.**

**
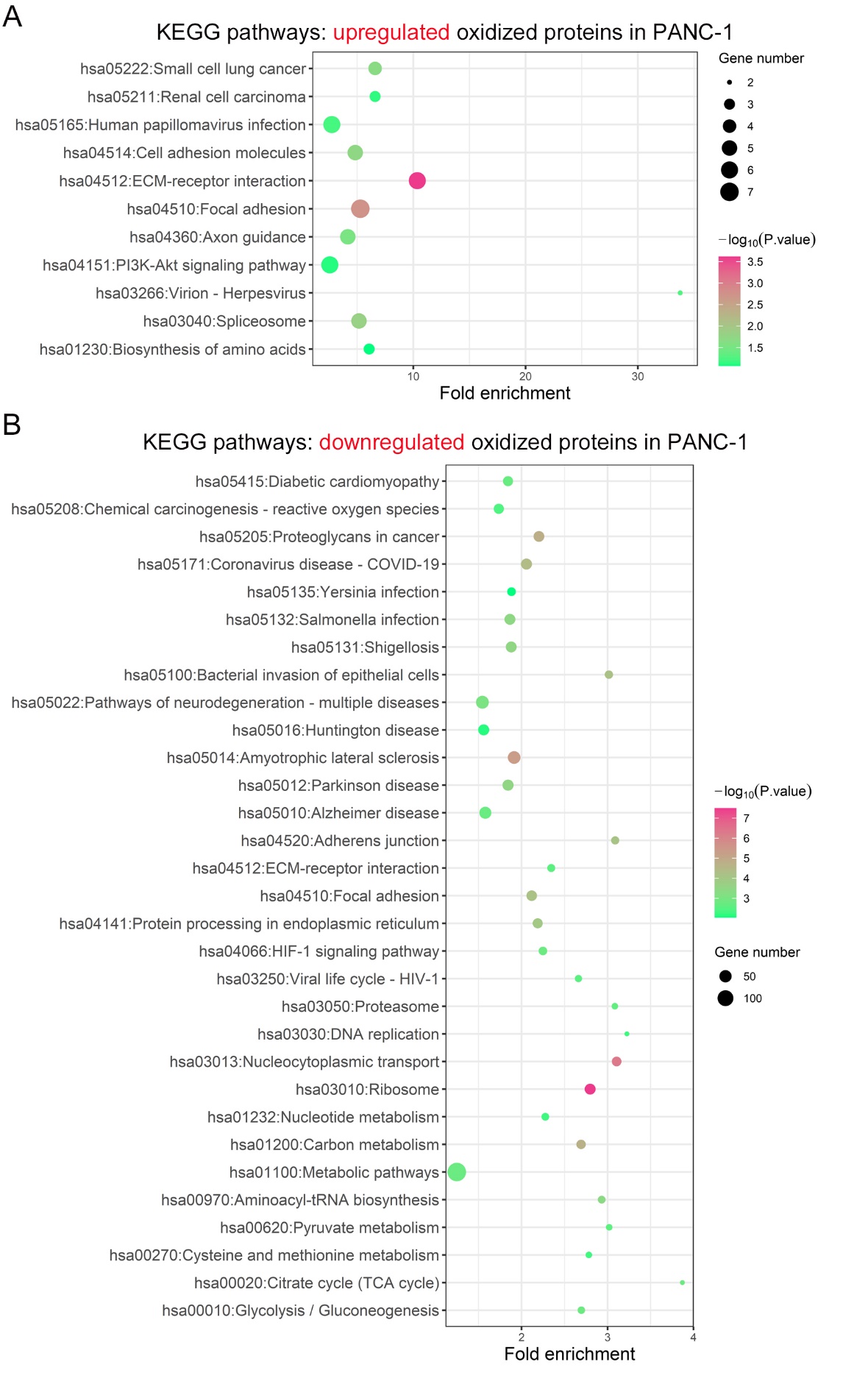
**

**Fig. S6. KEGG pathways with significant enrichment of oxidized proteins up-regulated or down-regulated in PANC-1 cells.**

**
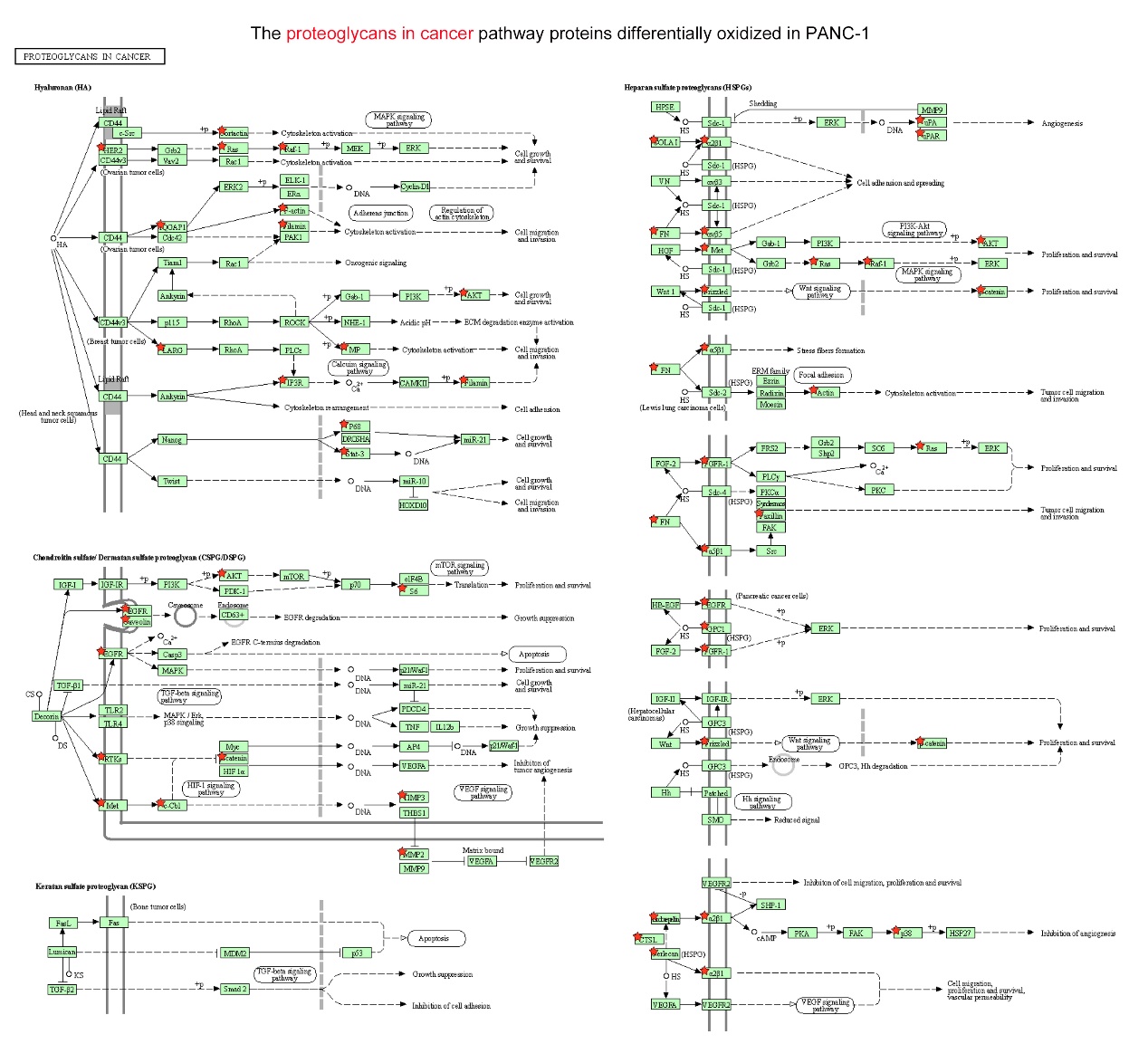
**

**Fig. S7. Oxidized proteins in the proteoglycans in cancer pathway.**

The schematic illustration of significant enrichment of oxidized proteins was modified from KEGG pathway database ([www.kegg.jp](http://www.kegg.jp)).

**
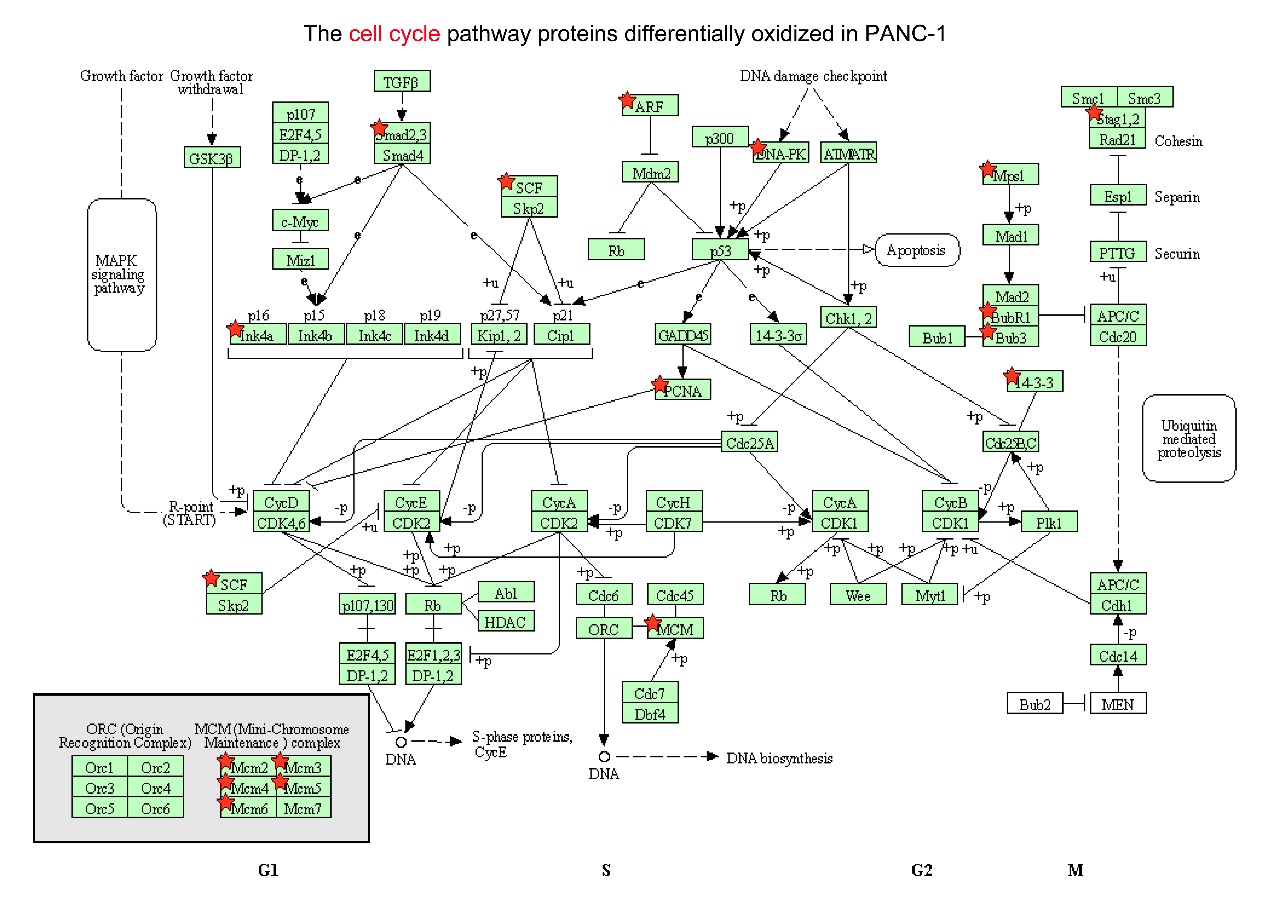
**

**Fig. S8. Oxidized proteins in the cell cycle pathway.**

The schematic illustration of significant enrichment of oxidized proteins was modified from KEGG pathway database ([www.kegg.jp](http://www.kegg.jp)).

**
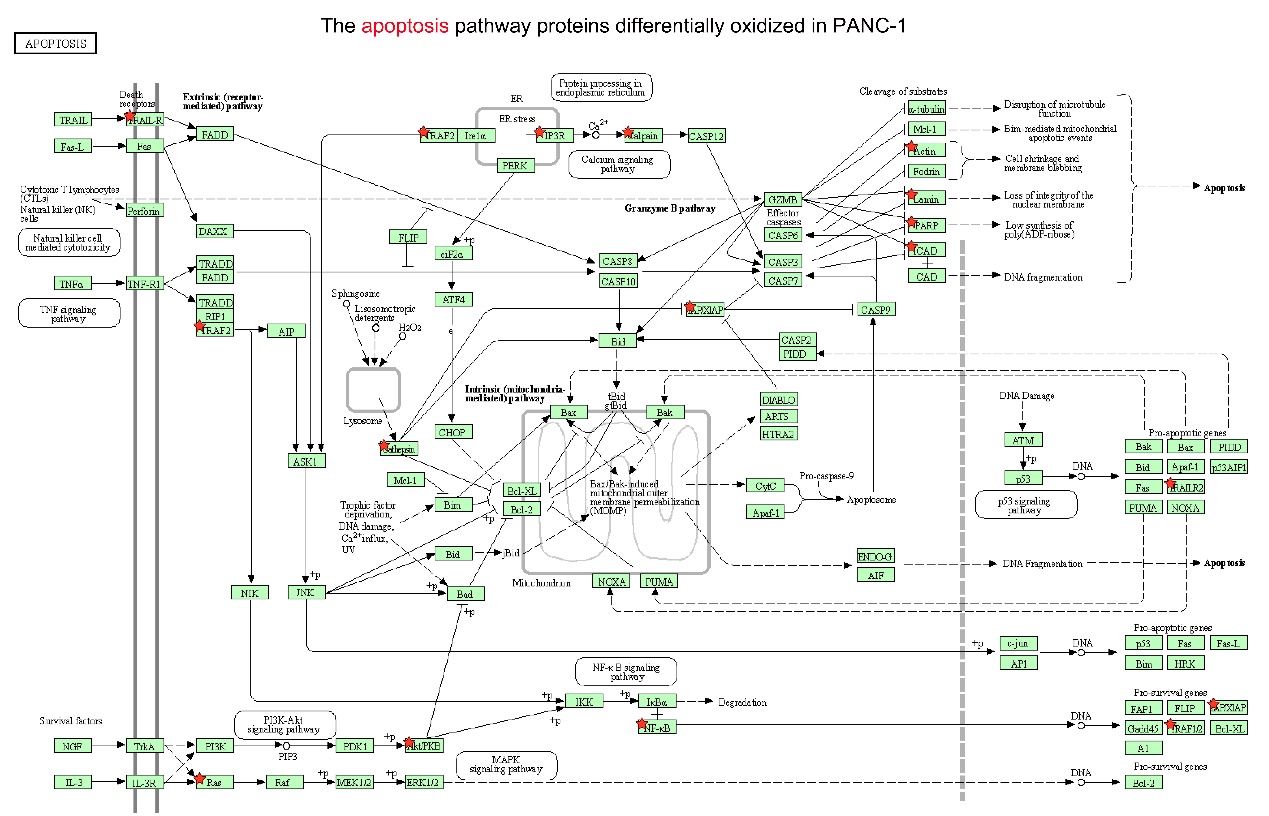
**

**Fig. S9. Oxidized proteins in the apoptosis pathway.**

The schematic illustration of significant enrichment of oxidized proteins was modified from KEGG pathway database ([www.kegg.jp](http://www.kegg.jp)).

**
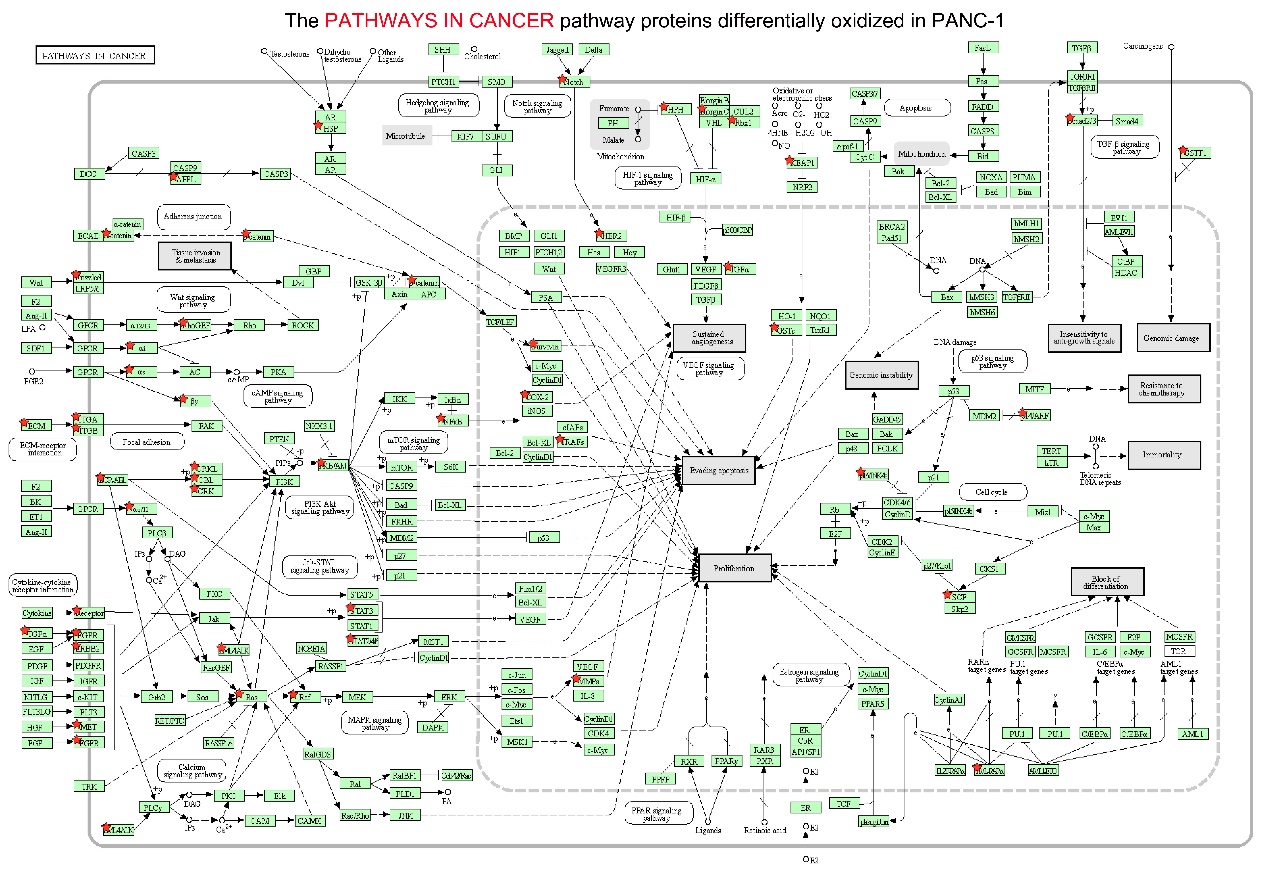
**

**Fig. S10. Oxidized proteins in the cancer-related pathways.**

The schematic illustration of significant enrichment of oxidized proteins was modified from KEGG pathway database ([www.kegg.jp](http://www.kegg.jp)).

**
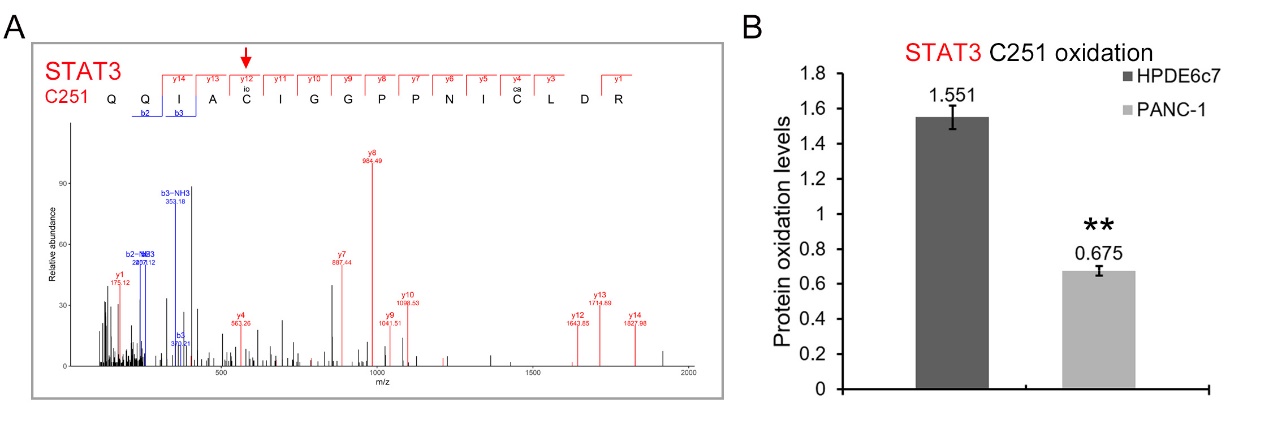
**

**Fig. S11. Decreased STAT3 oxidation in PANC-1 cells.**

The oxidation of Cys251 in STAT3 protein between HPDE6c7 and PANC-1 cells was identified by LC-MS/MS (A) and quantitated by label-free strategy (B).
